# Quantification of Blood Loss Improves Detection of Postpartum Hemorrhage and Accuracy of Postpartum Hemorrhage Rates: A Retrospective Cohort Study

**DOI:** 10.7759/cureus.13591

**Published:** 2021-02-27

**Authors:** Colleen Blosser, Alisha Smith, Aaron T Poole

**Affiliations:** 1 Obstetrics and Gynecology, Naval Medical Center Portsmouth, Portsmouth, USA; 2 Obstetrics and Gynecology, Naval Medical Center Camp Lejeune, Camp Lejeune, USA; 3 Obstetrics and Gynecology, Maternal-Fetal Medicine, Las Palmas Del Sol, El Paso, USA

**Keywords:** blood loss, postpartum hemorrhage, estimation, transfusion, quantitative

## Abstract

Objective

To evaluate the ability of estimated blood loss (EBL) and quantitative blood loss (QBL) to predict the need for blood transfusion in postpartum patients.

Methods

This is a retrospective observational study involving all deliveries one year before and after the change from EBL to QBL assessment in June 2017. Blood loss, need for blood transfusion, admission hematocrit, and postpartum nadir hematocrit were collected. Descriptive and bivariable analyses were performed. Receiver operator curves were compared.

Results

Overall, the baseline characteristics between the EBL (n=2743) and QBL (n=2,712) groups were similar. Although there was a higher rate of blood loss ≥ 1,000 mL in QBL vs EBL (6.5% vs 2.1%, P<0.001), there was no difference in the rate of blood transfusions (2.0% vs 2.0%, P=1). Among cesarean deliveries, QBL outperformed EBL for predicting blood transfusion and/or ≥10 point drop in hematocrit (AUC 0.75 vs 0.66, P=0.02). QBL also outperformed EBL for predicting transfusion after vaginal delivery (AUC 0.93 vs 0.81, P=0.03).

Conclusion

QBL is a more sensitive test for detecting clinically significant blood loss, which could lead to earlier recognition of hemorrhage and interventions.

## Introduction

Postpartum hemorrhage is the fourth leading cause of maternal mortality in the United States as well as the top most preventable cause, with an estimated 70% of maternal mortality cases being preventable. Chief among the reasons identified for preventable deaths due to hemorrhage was delayed recognition and treatment of significant blood loss following birth [1].

Visual estimation of blood loss (EBL) is known to be imprecise, with almost universal underestimation by 33%-50% in cases of high blood loss [2]. Given that signs and symptoms, including hypotension and tachycardia, often do not present until blood loss is substantial, underestimation of blood loss may be a major factor in the lack of timely identification and response to ongoing blood loss [3]. Therefore, leading national organizations have recommended quantitative blood loss (QBL) at every birth in order to recognize significant blood loss early and facilitate timely intervention [1,4-6].

Numerous studies have been conducted to evaluate the accuracy of different QBL methods, including photospectometry, collection bags or containers, weighed blood loss, and a combination of weighed blood loss and collection containers. Cumulatively, the evidence has demonstrated better accuracy with QBL versus EBL, particularly when blood loss is over 1,000 mL. However, there is limited evidence showing improved clinical outcomes; therefore, QBL has been criticized as an unnecessary, time-consuming, and resource-intensive process [7]. Despite national recommendations to implement QBL, most institutions continue to use EBL to evaluate blood loss due to concerns for cost and time involved with training and implementation to a quantitative assessment.

The aim of this study was to implement a protocol for QBL following all births and to compare blood loss, hematocrit changes, need for blood transfusions, and patient outcomes prior to and after the implementation of the QBL protocol.

## Materials and methods

In this retrospective cohort study, we analyzed births at a military tertiary care center from June 2016 to May 2018 to compare the predictive ability of EBL and QBL for postpartum hemorrhage. In June 2017, a quantitative approach to blood loss was implemented using a combination of directly measured blood loss and weighed blood loss. Nurses, obstetric providers, and anesthesia providers were instructed on the new protocol and goals. The institutional review board waived this quality improvement project for both its performance and publication.

Prior to the institution of QBL, the delivery provider, in conjunction with nursing and anesthesia, providers would provide an estimate of blood loss based on visual assessment. The QBL protocol for vaginal births required that the birth nurse and provider note the amount of fluid in a graduated under-buttocks drape immediately following the birth of the infant and prior to delivery of the placenta; this amount represented the amniotic fluid. The amniotic fluid amount was subtracted from the total amount recorded in the drape to determine the measured blood loss. The QBL protocol for cesarean births required that the surgical team suction the amniotic fluid from the surgical drape immediately following birth of the infant and prior to delivery of the placenta; the circulating nurse noted this amount in the suction canister, which represented the amniotic fluid. This amount was subtracted from the total amount in the canister to determine the measured blood loss. For both vaginal and cesarean births, all blood-soaked materials were weighed. The dry weights of the materials were subtracted from the total weight to determine the weighed blood loss. The measured blood loss was then added to the weighed blood loss to obtain the total QBL. When there was ongoing blood loss, the birth nurse provided regular updates on QBL amount to the provider. To help reduce mathematical errors and promote efficiency, a standardized QBL calculator containing all dry weights was introduced to the electronic health record. 

The electronic birth log was used to obtain data including demographic and birth information on all deliveries during the study from all consecutive births during the study period. Deliveries at less than 20 weeks gestational age were excluded. Route of delivery, multifetal gestation, and other risk factors were reviewed. Blood loss for each birth, admission hematocrit, postpartum nadir hematocrit for the hospital stay, and the transfusion of blood products were collected and organized in a Microsoft Excel spreadsheet for data analysis. Postpartum hemorrhage was defined as blood loss greater or equal to 1000 mL regardless of route of delivery. The decision to transfuse a patient was determined by the obstetric team. All patients admitted to labor and delivery had a pre-delivery complete blood count. All cesarean deliveries had a complete blood count 8 to 12 hours after delivery. In the EBL group, all vaginal deliveries had a complete blood count 8 to 12 hours after delivery while in the QBL group, a complete blood count was only ordered at the discretion of the obstetric provider if concerned about blood loss. The study size was based on a convenience sample of one year before and after implementation.

Documented blood loss was then compared to treatment with blood transfusion for all deliveries defined as having received at least one unit of packed red blood cells. QBL sensitivity and specificity for the prediction of blood transfusion were then calculated and compared to those of EBL and QBL. Results were stratified by birth type; for cesarean births, sensitivity and specificity for the prediction of hematocrit drop and/or postpartum blood transfusion were calculated and compared to those of EBL. If missing data could not be verified by individual chart review, those patients were removed from the study. All statistical analyses were performed using Prism (GraphPad Prism V 7, La Jolla, CA) or Excel (Microsoft). Chi-square or Fisher exact tests were used to analyze categorical variables as appropriate. Student t-test, Mann Whitney U, and Pearson correlation coefficient were used as appropriate to analyze continuous variables. A P value of < 0.05 and a 95% CI that did not cross 1 were considered statistically significant. Receiver operator curves were created to compare the accuracy of EBL and QBL.

## Results

There were a total of 5,454 deliveries in the initial analysis with 18 excluded due to delivery prior to 20 weeks (12 pre, 6 post). The EBL group consisted of 2,731 patients with 848 cesarean births (31.1%), and the QBL group consisted of 2,705 patients with 828 cesarean births (30.5%). Patient demographics were similar overall between the two groups with the exception of BMI and operative vaginal delivery rate (Table 1).

**Table 1:** Baseline demographics of the estimated blood loss group compared to the quantitative blood loss group. EBL, estimated blood loss; QBL, quantitative blood loss; BMI, body mass index. Values expressed as n (%), mean ± standard deviation. Missing values: maternal age (none), race (none), BMI (EBL 311, QBL 90), gest age at birth (EBL 0, QBL 1), birth weight (69 EBL, 74 QBL), nulliparity (none).

	EBL (n=2731)	QBL (n=2705)	P-value
Cesarean delivery	848 (31.1%)	828 (30.6%)	0.77
Operative vaginal delivery	96 (3.5%)	69 (2.6%)	0.04
Nulliparity	1331 (48.5%)	1287 (47.4%)	0.43
Race			0.06
White	1558 (57.0%)	1475 (54.5%)	
Black	673 (24.6%)	640 (23.7%)	
Asian	114 (4.2%)	122 (4.5%)	
Hispanic	40 (1.5%)	42 (1.5%)	
Other	386 (14.1%)	473 (17.3%)	
Maternal age at delivery	29.5 ± 5.2	28.7 ± 5.2	0.13
Maternal BMI at Birth (kg/m^2^)	31.5 ± 5.5	31.8 ± 5.6	0.04
Gestational age at birth (weeks)	38.9 ± 2.3	38.8 ± 2.3	0.06
Infant weight at birth (kg)	3.32 ± 0.78	3.32 ± 0.86	0.82

For cesarean births, the rate of patients with a significant hematocrit drop (≥ 10) and/or transfusion was additionally analyzed (Table 2).

**Table 2:** Transfusion and blood loss seen among all births (vaginal and cesarean deliveries). EBL, estimated blood loss; QBL, quantitative blood loss. Data presented as n(%).

	EBL (n=2731)	QBL (n=2705)	P-value
Transfusion of packed red blood cells	57 (2.0%)	56 (2.0%)	1
Tranexamic acid use	4 (0.1%)	184 (6.8%)	<0.001
Blood loss ≥ 500 mL	984 (35.9%)	842 (31.0%)	0.002
Blood loss ≥ 1,000 mL	57 (2.1%)	175 (6.5%)	<0.001
Blood loss > 1,200 mL	20 (0.7%)	96 (3.5%)	<0.001

There was a statistically higher rate of postpartum hemorrhage in the QBL group versus the EBL group (6.5% vs 2.1%, P<0.001). However, there was no difference in transfusion rate between the QBL group and EBL group (2.0% vs 2.0%, P=1). Subgroup analyses were completed to evaluate the performance of EBL versus QBL in vaginal and cesarean births. 

Vaginal births

**Figure 1:**
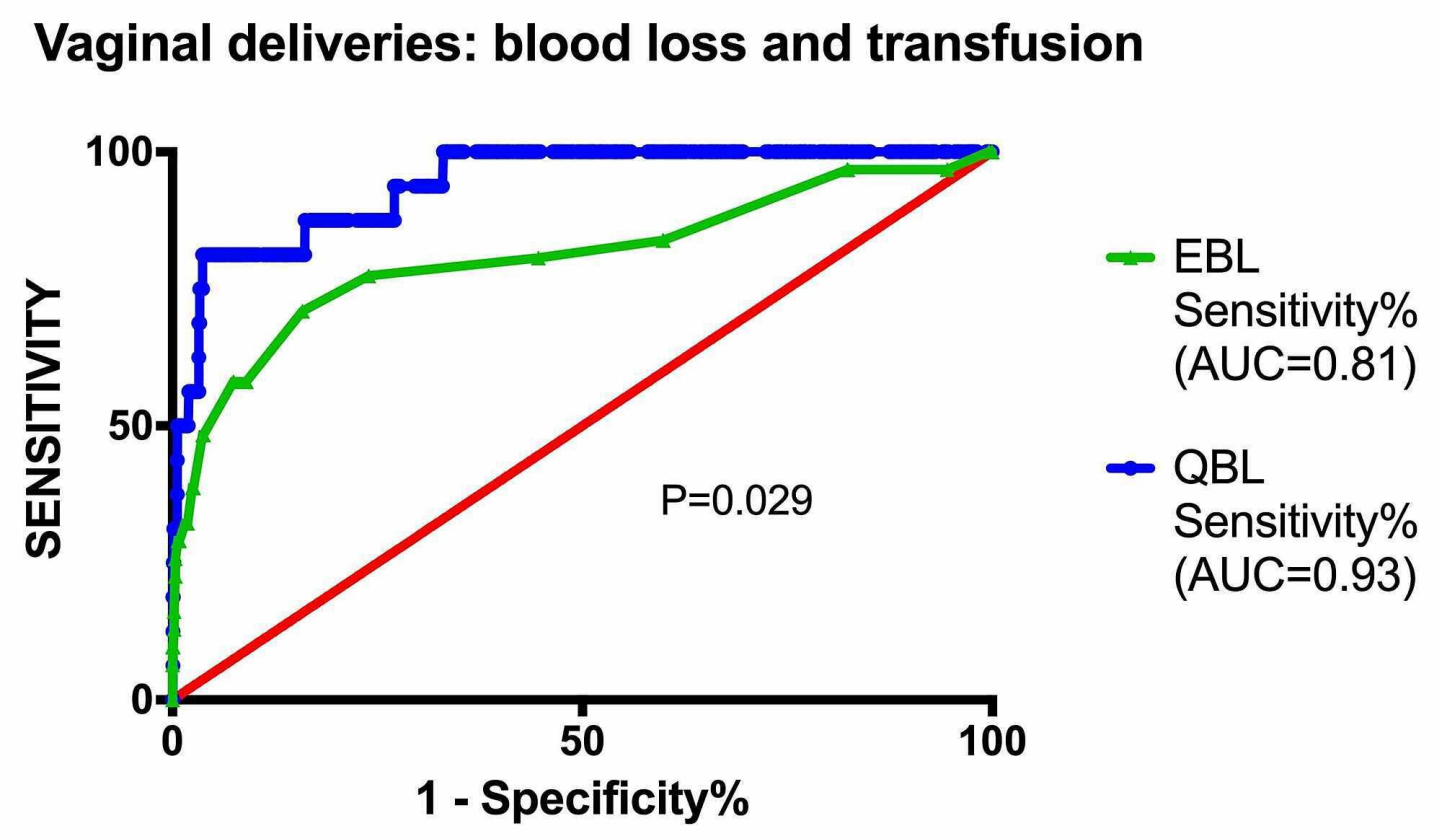
Comparison of EBL to QBL is predicting blood transfusion in vaginal births. EBL, estimated blood loss; QBL, quantitative blood loss; AUC, area under the curve.

Among vaginal births, the median recorded blood loss was statistically higher in the EBL group (250 mL with 95% CI of 200-250) compared to the QBL group (187 mL with 95% CI of 90-339). The 90th percentile for recorded blood loss was 400 mL for vaginal births with EBL compared to 526 mL for QBL. Conversely, the overall postpartum hemorrhage (blood loss ≥1,000 mL) rate was significantly higher in the QBL group vs the EBL group (1.9% vs 1.1%, P=0.046). Although there was not a significant difference in the blood transfusion rate between the EBL and QBL groups, there was a trend towards decreased transfusion in the QBL group (1.4% vs 0.9%, P=0.22). EBL was found to have greater specificity compared to QBL for predicting postpartum hemorrhage and blood transfusion (P<0.001). QBL demonstrated a trend for increased sensitivity when compared to EBL in predicting postpartum hemorrhage and blood transfusion (52.9% vs 30.8%), although this did not rise to statistical significance. Figure 1 shows EBL versus QBL in vaginal births on a receiver operator curve, which demonstrates the superior accuracy of QBL in the prediction of postpartum hemorrhage. The area under the curve (AUC) was found to be significantly higher (0.93 vs 0.81, P=0.03).

Cesarean births

In cesarean births, there was no difference in the median recorded blood loss between the EBL and QBL group (700 mL vs 660.5 mL, P=0.29). There was a significantly higher percentage of postpartum hemorrhage in the QBL group vs the EBL group (16.8% vs 4.2%, P<0.001). Additionally, the 90th percentile for recorded blood loss was much higher in the QBL group versus the EBL group (1201.8 mL vs 872 mL) However, there was no difference in transfusion rate (QBL 4.7% vs EBL 3.7%, P=0.33) and no difference in significant hematocrit drop and/or transfusion (QBL 14.0% vs EBL 13.7%, P=0.89). EBL showed a higher specificity for predicting postpartum hemorrhage with blood transfusion compared to QBL (96.7% vs 85.2%, P<0.001). However, QBL was more than twice as sensitive for the prediction of postpartum hemorrhage with blood transfusion compared to EBL (59.0% vs 29.0%, P<0.001).

**Figure 2:**
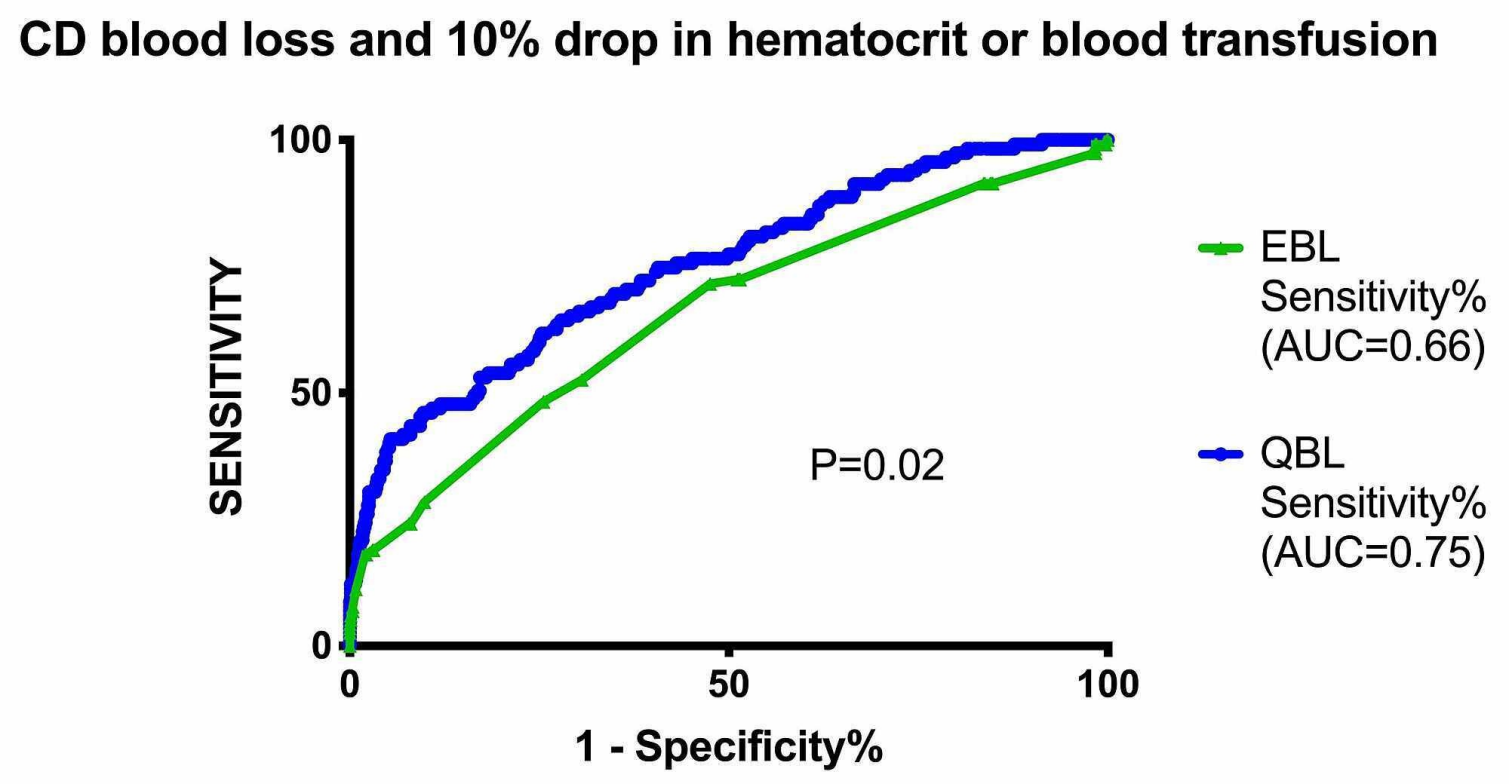
Comparison of EBL to QBL is predicting blood transfusion and/or ≥10 point drop in hematocrit in cesarean births. CD, cesarean delivery; EBL, estimated blood loss; QBL, quantitative blood loss; AUC, area under the curve.

Figure 2 depicts EBL versus QBL in cesarean births on a receiver operator curve, showing the superior accuracy of QBL in the prediction of postpartum hemorrhage. The area under the curve (AUC) was found to be significantly higher (0.75 vs 0.66, P=0.02). 

## Discussion

Our postpartum hemorrhage rate among the EBL group (2.1%) was consistent with other facilities in the United States participating in the National Perinatal Information Center reporting (NIPC). However, when QBL was implemented the rate of hemorrhage among vaginal and cesarean deliveries tripled to 6.5%. Specifically, the hemorrhage rate was 17% in cesarean births utilizing QBL and the 90th%tile of blood loss in this group was calculated to be 1,202 mL. These findings are consistent with prior studies. Katz et al. reported a 13% hemorrhage rate using QBL among cesarean deliveries with the 90th %tile approaching 1,400 mL [8]. 

Despite the higher rates of postpartum hemorrhage in our study, there was no difference in the rate of blood transfusions. In cesarean births, there was also no difference in the rate of significant drop in hematocrit. This suggests that although the documented hemorrhage rate was higher among the QBL group, actual blood loss was likely comparable between the two groups. 

The extraordinarily high PPH rate among the QBL group suggests that QBL could be an overestimate, EBL an underestimate, or both a combination of overestimation of QBL and underestimation of EBL. Regardless, if the definition of postpartum hemorrhage is to remain the same (1,000 mL) for cesarean births when QBL is utilized, then the anticipated hemorrhage rate should be expected to be substantially higher. Furthermore, consideration should be taken in redefining postpartum hemorrhage with cesarean delivery to greater than 1,200-1,400 mL (90%tile) if QBL is utilized. Prior studies have found that a QBL of 1,522 mL predicted the need for blood transfusion with a sensitivity of 74%, indicating that a cutoff of 1,200-1,400 mL would have a higher sensitivity [9]. Further analysis is needed to determine the optimal definition of PPH while utilizing quantitative measures of blood loss.

QBL was found to be more sensitive in detecting postpartum hemorrhage with the need for blood transfusion and/or significant hematocrit drop. Our findings reinforce those from previous studies showing that QBL is a more accurate measurement of blood loss, suggesting that QBL may be of important clinical significance in proactively identifying patients who require blood transfusion or additional monitoring based on blood loss, prior to the onset of symptoms.

Data from the sub-analysis of vaginal births suggest that providers tend to overestimate the average blood loss associated with a vaginal birth. This is in concordance with findings published by Hamm and colleagues [10]. However, the documented hemorrhage rate among the QBL vaginal birth sub-group was significantly higher than the corresponding EBL group (1.9% vs. 1.1%, P<0.05). Despite a higher postpartum hemorrhage rate, there was a trend toward a decrease in blood transfusions. This finding may be related to the significantly higher TXA usage (0.05% in the EBL group vs. 3.5% in the QBL group) or a result of earlier interventions, including TXA administration, resulting from QBL utilization.

Our study is not without limitations. The studied population consists only of active duty military members and their dependents, which may be healthier overall than that seen in civilian tertiary care centers and also have reliable access to no-cost medical care. 

The policy and practice regarding the use of tranexamic acid was updated during the study period. Prior to January of 2017, tranexamic acid use was rare and limited to patients with bleeding disorders. In January 2017, a new policy was instituted to consider the administration of 1 gram of tranexamic acid over 10 minutes when blood loss exceeded 500 mL in vaginal births or 1,000 mL in cesarean births. This change in practice corresponded with an increased use of tranexamic acid in the QBL group as compared to the EBL group. This increased use may have impacted total blood loss. 

No data was collected regarding the frequency and use of other medications or modalities for the prevention and treatment of postpartum hemorrhage. However, there was no difference in either transfusion rates or ≥ 10 point hematocrit drop between the EBL and QBL groups, which indicates probable similarity in actual blood loss.

Finally, national organizations recommend QBL based on maternal mortality reviews. While a recent study published in 2019 showed increased use of uterotonics and earlier time to blood transfusion with implementation of QBL, there has not been any studies to date showing an improvement in mortality or volume of blood transfused with the use of quantitative blood loss [8]. It is unlikely, even in large studies such as ours, that the full clinical benefit of QBL can be accurately captured, particularly as it pertains to maternal mortality. Although we have experienced a handful of cases where QBL was the only factor used to initiate interventions in response to postpartum hemorrhage and may have initiated life-saving measures, these cases were not captured in our data analysis.

## Conclusions

QBL is a more sensitive test for detecting clinically significant blood loss, which could lead to earlier recognition of hemorrhage and more timely intervention. The implementation of QBL results in higher documented hemorrhage rates without concurrent increases in transfusions or a significant drop in hematocrit. This is particularly true among Cesarean births, given that the 90th percentile of QBL (1,200 mL) far exceeds the definition of postpartum hemorrhage (1,000 mL). The authors advocate for the utilization of QBL due to its increased sensitivity. However, when comparing hemorrhage rates to other facilities or national averages, the difference in the mode of blood loss assessment must be considered, and facilities using EBL should not be compared to those using QBL.
